# A rare case of closed total talar dislocation on a background of ankle instability

**DOI:** 10.1016/j.radcr.2025.07.038

**Published:** 2025-08-08

**Authors:** Rashed Al-Khudairi, Ruhaid Khurram, Alexandros Maris, Angelo Vasiliadis

**Affiliations:** a Department of Radiology, Royal Free Hospital NHS Trust, London NW3 2QG, UK; b Department of Trauma and Orthopaedics, Royal Free Hospital NHS Trust, London NW3 2QG, UK; c Department of Orthopaedic Surgery, St Luke’s Hospital, Panorama, Greece

**Keywords:** Total talar dislocation, Pan talar dislocation, Luxatio tali totalis

## Abstract

Total talar dislocation is a rare and potentially devastating ankle injury accounting for 3.4% of all major talar injuries and is often associated with high-energy impact. There is tri-articular dislocation involving the subtalar, talonavicular, and tibiotalar joints. The injury can be open or closed and with or without co-existent fractures. Surgical management can be challenging. We present a rare case of total talar dislocation in a middle-aged male with classic radiographic appearances, pre-existing ankle instability as a risk factor, which was successfully managed with closed reduction. We discuss the key considerations in the radiological and surgical follow-up of these cases.

## Introduction

Total talar dislocation, also known as pan talar dislocation and luxatio tali totalis, is a very rare and potentially devastating ankle injury accounting for 3.4% of all major talar injuries [1]. This significant injury is associated with high-energy impact, typically from a road traffic accident or fall from height, where there is a combination of tibiotalar plantarflexion and subtalar supination or pronation.

This can be diagnosed on plain radiographs, where there is talar extrusion and tri-articular dislocation. The pattern of dislocation has been described involving initially the subtalar joint, followed by the talonavicular joint, and then the tibiotalar joint [2]. The injury can be closed or open, with and without co-existent fractures. Given the infrequency at which this injury occurs, there is debate with regards to the ideal treatment strategy to minimize complications and reduce morbidity.

## Case report

A middle-aged male presented to the emergency department with an acutely deformed right foot. The patient was intoxicated at presentation and recalled sustaining an injury; however, could not provide further information on the mechanism of trauma. He was a smoker with a previous lateral malleolus fracture and history of ankle instability.

On examination, the right foot was deformed at the Chopart joint, and motor function was impaired. Vital signs were normal. There was no neurovascular compromise, and the injury was closed. No additional injuries were identified.

**Fig. 1:**
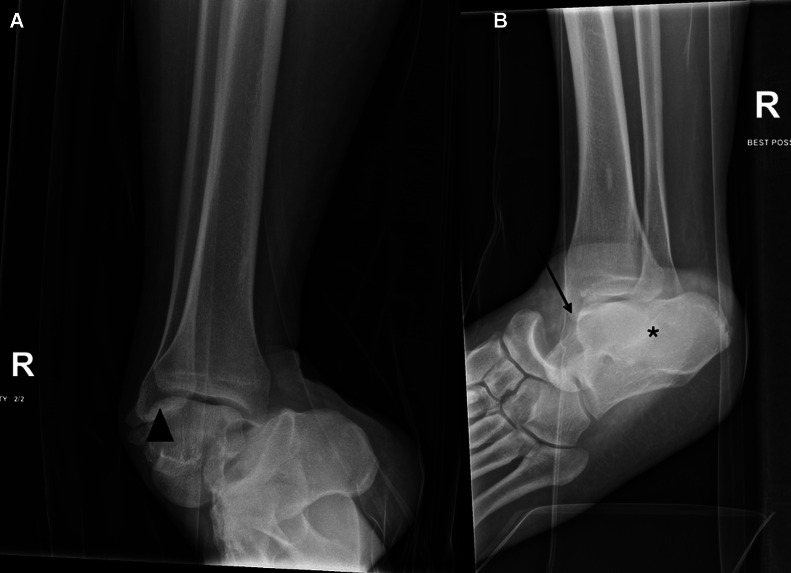
Best possible anteroposterior (A) and lateral (B) radiographs of the right ankle. There is tri-articular dislocation of the talonavicular (arrow), subtalar (asterisk), and tibiotalar (arrowhead) joints.

Best possible AP and lateral radiographs of the right ankle were obtained (Fig. 1), which demonstrated tri-articular dislocation involving the talonavicular, subtalar, and tibiotalar joints.

**Fig. 2:**
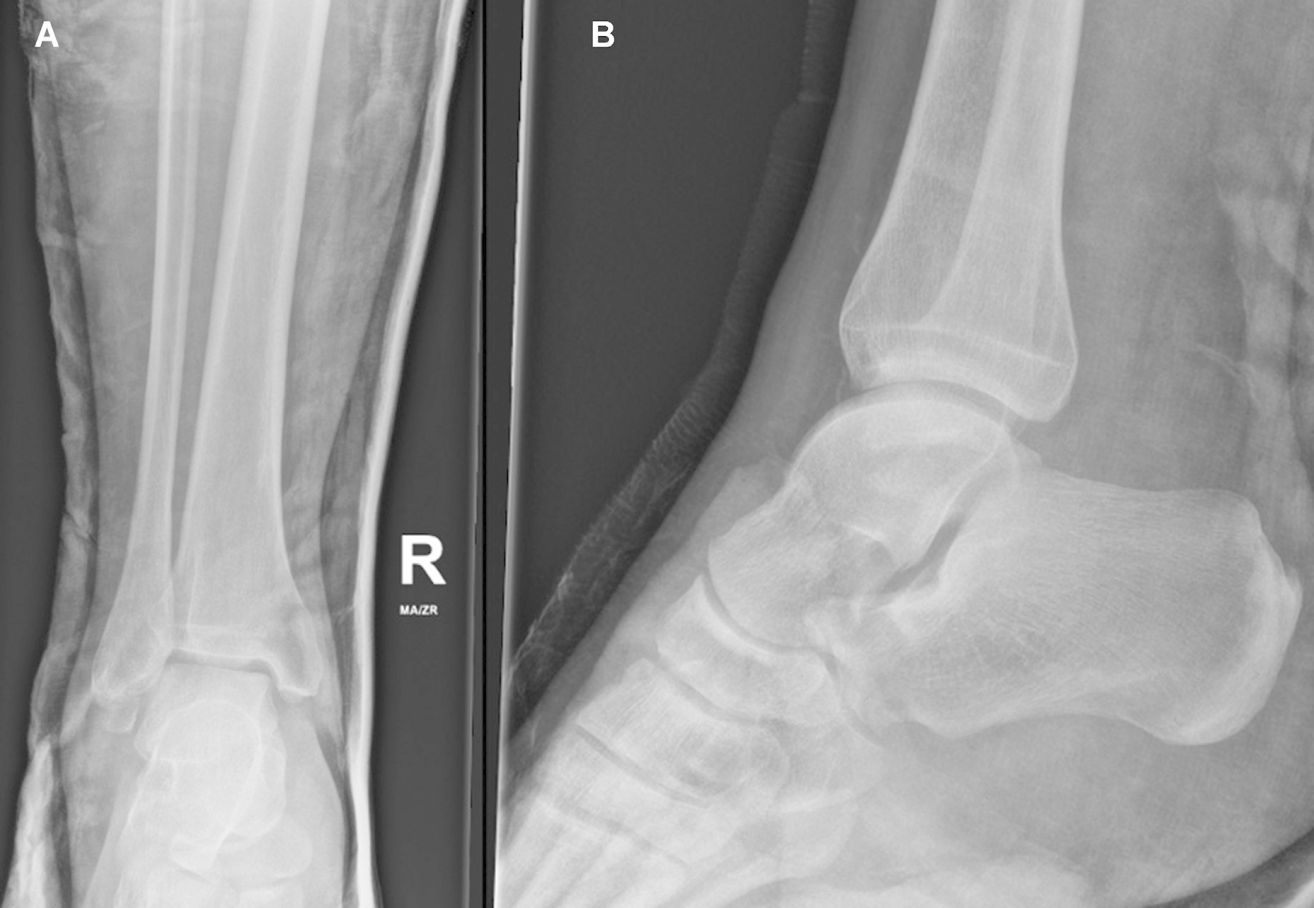
Postreduction anteroposterior (A) and lateral (B) radiographs of the right ankle. There is satisfactory alignment at the talonavicular, subtalar, and tibiotalar joints.

Subsequently, the patient had a closed reduction under sedation, followed by application of a below-knee back slab. Repeat postreduction radiographs (Fig. 2) showed satisfactory tri-articular alignment with no acute fracture. There was a well-corticated bone fragment adjacent to the lateral malleolus, which was present on prior radiographs, in keeping with an old injury.

**Fig. 3:**
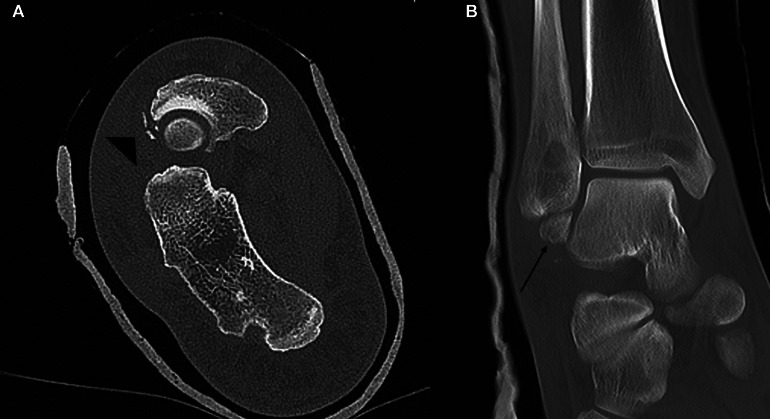
Postreduction axial (A) and coronal (B) CT slices of the right ankle. There are small avulsed bone fragments from the lateral aspect of the navicular (arrowhead). There is a well-corticated bone fragment inferior to the lateral malleolus in keeping with an old fracture (arrow).

A CT scan was performed to assess for occult fractures (Fig. 3). There were tiny avulsed bone fragments arising from the lateral aspect of the navicular bone; however, no talar or malleolar fractures were noted.

**Fig. 4:**
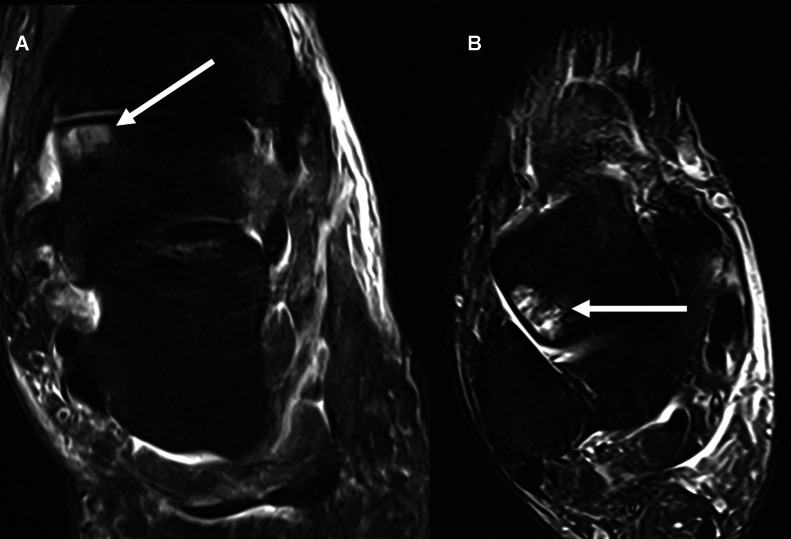
Postreduction coronal (A) and axial (B) proton density fat-saturated MRI sequences of the right ankle 2 weeks after the acute injury. There is a subtle impaction injury of the medial talar head articular surface with associated underlying marrow oedema like signal (white arrows).

The patient was discharged with crutches and advised to remain nonweight-bearing on the right lower limb. Two weeks after discharge, the patient reported persistent soft tissue swelling and pain for which an MRI was performed (Fig. 4). On the MRI, there was a shallow impaction injury of the medial talar head articular surface with underlying mild marrow oedema like signal; however, no evidence of osteomyelitis or osteonecrosis.

Eight weeks following the injury, the cast was removed and the patient was able to walk without complications.

## Discussion

The position of the talus is primarily maintained by ligamentous structures, including the deltoid ligament, anterior/posterior talofibular ligaments, and the interosseus ligament. The mechanism of injury of talar dislocation typically occurs with the foot in plantarflexion with either pronation or supination [3]. Anterolateral dislocation is most common (>50% of cases), however, anteromedial and posteromedial dislocation can also occur [4]. The high-energy trauma and axial load in combination with forced plantarflexion and rotational stress, initially causes subtalar dislocation, followed by dislocation of the talonavicular and tibiotalar joints [2].

There are several factors that may put patients at increased risk for this rare injury. These include ligament laxity around the ankle joint, peroneal muscle weakness, and repeated ankle sprains [5]. In our case, the patient had a history of ankle instability, and on the plain radiograph, there was a well-corticated bone fragment adjacent to the lateral malleolus, in keeping with an old fracture.

CT has several advantages over plain radiographs in this context. Due to the overlap of bony structures on radiographs, subtle or nondisplaced fractures can often be missed [6]. CT allows better evaluation of displacement, comminution, and articular involvement. For instance, the tiny avulsed bone fragments arising from the navicular were not see on the radiographs but easily appreciated on the CT. Additionally three-dimensional reformatted images improve understanding of fracture morphology and extent, which in turn will facilitate surgical decision making and preoperative planning [6].

Osteoarthritis, osteomyelitis, and osteonecrosis are significant complications that can be assessed on MRI. Osteomyelitis and osteonecrosis can occur in the early phase and are caused by damage of the talar blood supply and adjacent soft tissues. This can have a significant impact on long-term prognosis [1]. Osteonecrosis of the talus has the same MRI findings as osteonecrosis in other parts of the body. Early findings include the “double line sign” on T2-weighted images with peripheral low and inner high signal intensity lines. Delayed findings include subchondral collapse with associated joint effusion and synovitis [7]. Key MRI features of osteomyelitis include bone marrow oedema, cortical disruption, bone enhancement, and associated soft tissue findings, which include soft tissue oedema, sinus tract formation, and fluid collections [8]. Osteoarthritis is a late complication that can be confirmed on plain radiographs with classical degenerative appearances of joint space narrowing, subchondral cysts, subchondral sclerosis, and osteophyte formation.

Treatment depends on several factors, including open vs closed injury, neurovascular compromise, and concomitant fractures. 85% of these injuries are open and 15% are closed [9]. Pure pan talar dislocations without fractures have been frequently reported in the literature [[10], [11], [12]]. In this case, there were small avulsed bone fragments from the lateral aspect of the navicular, however, no acute fractures of the talus, medial malleolus, and lateral malleolus on plain radiographs or CT. There was a subtle impaction injury at the talar head on the MRI.

Management of pan talar dislocation is complex, with no consensus on the ideal treatment. Talar reimplantation is an option, either via closed manipulation under anaesthesia (as in this case) or open reduction with/without fixation. Some authors suggest open reduction [13], however, closed reduction for closed dislocations has also been strongly suggested to reduce risk of soft tissue injury and damage to the vascular networks [14]. Primary talectomy and tibiocalcaneal arthrodesis is also performed in certain cases.

## Conclusion

Total talar dislocation has a classical tri-articular dislocation pattern that can be readily identified on plain radiographs. Ankle instability is a known risk factor. CT assessment is crucial to identify co-existent fractures, which may be occult on radiographs. MRI plays a role in early detection of significant complications, which include osteonecrosis and osteomyelitis. Surgical management is challenging; however, closed reduction alone can successfully manage the injury as seen in our case.

## Patient consent

Verbal and written informed consent for the publication of this case report was obtained from the patient.
